# The Gold Standard and the Pyrite Principle: Toward a Supplemental Frame of Reference

**DOI:** 10.3389/fpsyg.2020.00562

**Published:** 2020-03-31

**Authors:** Stanley L. Brodsky, Bronwen Lichtenstein

**Affiliations:** ^1^Psychology Department, The University of Alabama, Tuscaloosa, AL, United States; ^2^Department of Criminology and Criminal Justice, The University of Alabama, Tuscaloosa, AL, United States

**Keywords:** gold standard, gold history, construct validity, interpretations of gold standard, pyrite principle

## Abstract

In medicine and social sciences, the phrase “gold standard” is often used to characterize an object or procedure described as unequivocally the best in its genre, against which all others should be compared. Examples of this usage are readily available in rigorously peer-reviewed publications, touted by test publishers, and appear in descriptions of methodologies by social science researchers. The phrase does not accurately describe commonly accepted measures, tests, and instruments. Instead, the descriptor can be ambiguous and misleading. This paper presents an overview of the history of the gold standard and its current applications to medicine and the social sciences. We question the use of the phrase “the gold standard” and suggest the additional operational use of a “pyrite principle” as a less presumptuous frame of reference. In thinking about validity and standards, the pyrite principle permits an understanding of standards as authoritative rather than fixed constructs in behavioral and health sciences.

## Introduction

In this paper, we provide an initial examination of the nature of gold, how it links to the concept of the gold standard, and applications of this standard within the scientific realm. We critically consider specific applications of this benchmark to the social and health sciences, and then propose the pyrite principle as a vehicle for rethinking the gold standard in these areas operationally, but not necessarily to be presented in publications. Because the development of the gold standard as an expression of high value has not followed a linear path, we begin with a brief history of gold in human civilization.

## The Triumph of Gold

Gopher et al. (1990) are among the many anthropologists who wrote about the cultural symbolism of gold artifacts in Middle Eastern cemeteries as long as 6000 years ago. These authors cite reports of gold found in Paleolithic caves, although there is no clear evidence that gold was then valued or more than coincidently present at these sites. In fact, ancient cultures did not always consider gold desirable for currency, adornment, or applications in metallurgy. Some eras in human history have prized silver more highly, including at the time of the Roman Empire.

Overall, though, gold has been highly valued: but why? Rowlatt (2013) compared gold ore to silver, iron, potassium, and many other elements in the periodic table. He concluded that gold has two compelling traits. It is chemically unreactive, so that gold found thousands of years ago has stayed much the same over millennia. Gold is also relatively scarce but not truly rare and, compared with many other valuable elements, not difficult to extract from the earth’s silicate mantle. Gold’s chemical properties may be contrasted to those of, say, platinum, which has a significantly higher melting point and is denser. Platinum is more difficult to extract, a fact that partially explains its higher cost than gold ore. It is also the reason that gold gained dominance in common currency and, later, as a cultural symbol for objects of great value (Fohr, 2017). For currency purposes, the most common comparison of gold is to silver as a precious metal used for coinage. Indeed, both gold and silver have had historic monetary purposes for millennia. Silver, however, lost ground in 1717 when Sir Isaac Newton, master of Britain’s Royal Mint, adopted the gold standard in order to reduce counterfeiting and to elevate the status of his office (Belenkiy, 2013). Parenthetically, Newton also developed the concepts of standard deviations and sampling for assessing weight of the British guinea, a coin containing one-quarter ounce of gold that was minted between 1663 and 1814.

## The Origin of the Phrase “The Gold Standard”

One major linguistic source, StackExchange’s (2016) *English Language and Usage* offers two equally tenable origins of the phrase “the gold standard.” One is conceptual in which a social consensus develops around the perceived value of objects or procedures, and the element with the best quality earns the label of gold-like. The second origin is the literal gold standard as the conventional measure for currency conversion in global markets. In historical terms, financial markets determined the value of a new currency by comparing it to a standard quantity of gold for trading purposes (Claassen, 2005). The 18th century markets did not, as in modern times, use the gold standard to “mean standard of excellence” or “standard of perfection.” Indeed, Claassen noted that the market debated the usefulness of the gold standard until adopting a better system of currency valuation.

This etymological discussion segues into the emergence of the term “gold standard” in medical contexts, which appears to apply to social science and physical scientific contexts as well. The diffusion of this term into popular discourse, and into positivistic disciplines such as medicine and science, signals its symbolic triumph, even though the gold standard itself no longer dominates global financial markets. In the scientific arena, the descriptor “gold” is typically a synonym for “best of its kind,” much like the first definition above. For the present purposes, the simple phrase “best of its kind” has less of the absolutism and intense cognitive impact that comes about from interpreting the “gold standard” as the acme of perfection.

## The Gold Standard and Social Science

The term “gold standard” made its way into the social science literature in the twentieth century, following the example of medical science. After Rudd’s (1979) editorial in the *Archives of Internal Medicine* addressed the absence of a gold standard by which to measure patient compliance, the term appeared more often and was applied more broadly in psychology and other social sciences. This disciplinary expansion prompted Lilienfeld et al. (2015) to issue this caution to mental health professionals about using such problematic terms: “We present a provisional list of 50 commonly used terms in psychology, psychiatry, and allied fields that should be avoided, or at most used sparingly and with explicit caveats” (p.2). Lilienfeld et al. referred to “the gold standard” in the popularized (rather than intended sense) as the antithesis of the fallibility principle and faulted scientists who used it. The authors summarized their concerns as follows:

In the domains of psychological and psychiatric assessment, there are precious few, if any, genuine “gold standards.” Essentially all measures, even those with high levels of validity for their intended purposes, are necessarily fallible indicators of their respective constructs […]. As a consequence, the widespread practice of referring to even well-validated measures of personality or psychopathology, such as Hare’s (2003) Psychopathy Checklist-Revised, as “gold standards” for their respective constructs (Ermer et al., 2012), is misleading (see Skeem and Cooke, 2010). If authors intend to refer to measures as “extensively validated,” they should simply do so (Lilienfeld et al., 2015, p. 4).

## Psychology and Psychological Assessment

Substantial misuses of the phrase the gold standard in terms of tests and measurements have entered the vocabularies of professional and scholarly psychology. In this context, it should be noted that construct validity of tests is never a simple matter. In the Standards for Educational and Psychological Testing, the American Educational Research Association et al. (2014), with its sibling associations, stated there is rarely if ever a single valid meaning or interpretation that can be attached to a test score (p. 11). Instead, 25 distinct standards for validity of tests have been identified, including normative samples, relationships with other variables, and interpretations of effect size measures.

Following this general starting point, we look at the Wechsler Adult Intelligence Scale (WAIS; Wechsler, 1955), and its revisions, which have proven a popular and useful measure for over six decades (Weiss et al., 2010). In a detailed review, Hartman (2009, p. 85) praised the most recent edition of the WAIS (WAIS-IV; Wechsler, 2008) as the “return of the gold standard.” This claim bears scrutiny in terms of how the WAIS compares to other measures in the field.

First, the original WAIS and subsequent revisions developed using one of many theories of intelligence. The less popular Stanford-Binet measure of intelligence is the product of a competing theory that is not clearly less useful, or less-validated. Indeed Salekin et al. (2014) have reported high correlations between the two tests, high convergent validity, and cogent findings that both tests measure the same construct. Furthermore, the phrase “gold standard,” as used for psychological variables such as intelligence, may not apply to complex social constructs that vary by population, setting, and context. The Stanford-Binet 5 yields IQ scores extending over a wider range of intellectual functioning, providing an additional basis to question the common assignment of the gold standard to the WAIS-IV.

The Psychopathy Checklist Revised (PCL-R; Hare, 2003) is another psychological measure often mislabeled as “the gold standard,” in this case for measuring psychopathy (Vitacco et al., 2005). Psychopathy manifests in traits such as lack of anxiety, callousness, antisocial or irresponsible conduct, and manipulative behaviors. Although the PCL-R is unquestionably the most commonly used measure of psychopathy, scholars have raised major issues about its applications and limitations (Skeem and Cooke, 2010; Cox et al., 2013; Murrie et al., 2013). For individuals to obtain a high score on the measure (indicating they are highly psychopathic), they must meet antisocial and criminal behavior criteria (Forouzan and Cooke, 2005; Blackburn, 2007). Contention arises over exactly what the PCL-R measures, which in turn raises issues about whether the descriptor of gold standard, as currently used, is appropriate for PCL-R based assessments of psychopathy.

The marketing of psychological measures also draws on the phrase “gold standard,” occasionally with an awareness of the limitations of the descriptor. For example, Maggi M. Reiss, president of IDS Publishing, wrote that the Reiss Motivation Profile of Developmental Disabilities, “…represents the ‘gold standard’ in its field.” The quotation marks used by Reiss suggest a qualified use of the phrase. Nevertheless, it is clear that that use of the phrase is an example of marketing hyperbole rather than objective evaluation of the Reiss Motivation Profile for diagnostic purposes.

The Diagnostic and Statistical Manual of Mental Disorders, Fifth Edition (DSM-5; American Psychiatric Association, 2013) is another example of how “gold standard” is used in the mental health field. After examining state and federal case law, Perlin (2015) reported that expert witnesses regularly refer to the DSM-5 as the gold standard for diagnosing mental illness. Although the DSM was characterized for decades as the gold standard, some scholars (e.g., Pickersgill, 2014; Browne, 2017; Bachem and Casey, 2018) have argued that the diagnostic reliability of the DSM-5 was compromised by problems in construct validity and simplistic conceptualizations of diagnoses. Rudd’s (1979) call to keep searching for a gold standard in medical science fits with a more practical view of the DSM as one of the best taxonomies currently available for diagnosis, and recognizes the difficulty of establishing validity for all conditions and contexts.

An additional issue in characterizing the DSM-5 as the gold standard for diagnostic purposes concerns the alternative International Statistical Classification of Diseases and Related Health Problems, 10th Revision, Clinical Modification (ICD-10-CM). The ICD-10-CM classifies diagnoses, symptoms, and procedures in the health sector. The DSM-5 only includes psychological disorders, while the ICD-10-CM includes similar diagnoses as well as other medical conditions. The existence of both classification systems poses this question: in one area of knowledge, can there be contemporaneous “gold standards”?

## The Gold Standard in Experimental and Applied Social Science

The gold standard appears in two guises in descriptions of programmatic treatment research, either as a taken-for-granted standard for behavioral interventions, or as the basis for critiques when these interventions fail to live up to expectations. In both cases, the meaning of the gold standard implies a perfection unattainable in real-world interventions. For example, Padian et al. (2010) reviewed 36 randomized control trials (RCTs) for information of whether male circumcision, vaccination, and antiretroviral drug therapy prevented HIV transmission to sexual partners. The authors reported “flat” results, which they attributed to poor adherence or inadequate design and implementation of the standardized protocol. Poetically, they joined in the frequent and desirable embrace of multimethod approaches that offer “the strength and affordability that make alloys like steel so useful and durable” (p.10). Padian and colleagues issued a caution over gold standard assumptions in experimental social science, and recommended an “alloy” of multiple methods and lines of evidence in specific guidelines for HIV prevention. In the same sense, Chesney (2006) encouraged thinking of research methods as an adaptive rather than perfectionist model.

It may well be that reconceptualizing RCT standards is a path toward promoting and accepting positive elements of design and methodology, rather than applying the gold standard label to behavioral interventions. For example, Najavits (2015) wrote that questions about rates of retention in lauded treatment programs for Post-Traumatic Stress Disorder (PTSD) mean that claims of superiority for one PTSD therapy over another ring hollow, unless they account for the real-world conditions that drive rates of retention.

This critique of common uses of the gold standard may apply equally to evidence-based interventions. For example, Bovaird (2014) argued that macro-level policies derived from “best evidence” behavioral interventions (say, for obesity reduction or smoking cessation) are likely to fail if policymakers assume that the one-size-fits-all model accommodates all groups and conditions. For this reason, Bovaird asserted, “a gold standard may turn out (as in economics) to be a serious handicap in policy-making” (p. 20). A return to conceptualizing the gold standard as a flexible model in continuing evolution (Duggan, 1992) would address the fallibility of one-size-fits-all policies in public health planning.

In his presidential address to the American Society of Criminology, Clear (2010) voiced concerns about the value placed on randomized field trials (RFTs) (akin to the RCT) as the gold standard in criminology. Clear challenged the one-size-fits-all model for his discipline and voiced objections to RFTs as the gold standard. Clear concluded that the RFT standard is “more of a copper standard than gold,” because it appears to perpetuate certain standardized protocols or criteria that frustrate the need for a more nuanced approach to behavioral interventions.

Hough (2010) and Sampson (2010) voiced similar concerns about rigidly experimental criminology. They advocated the use of both qualitative and quantitative approaches to identify effective ways to prevent crime because, “these questions [about why people offend and how to stop it] are much more complex that those about the impact of pharmaceutical treatments” (Hough, 2010, p. 19). Hough concluded that no one method has proven better in predicting, controlling, or preventing criminality. A similar conclusion was reached by Sampson (2010) who declared “Criminologists should dispense with the use of ‘gold standard’ language (even in quotes!), and get on with the hard business of doing good research” (p. 499).

## Multiple Meanings of Gold Standard

To this point, our exploration of the gold standard has proceeded as if it presented a unitary concept, and as if there is a clear and shared understanding of its meaning. We began with this approach because the modern use of “gold standard” often appears as an inflexible approach to scientific research. Although some common usages are present, there are perhaps five different possible meanings of the gold standard in the social and behavioral sciences. The following list is preliminary, but these meanings seem to stand out for frequency and relevance.

1. As praise. In this usage, calling a test or procedure the gold standard may be a simple scholarly affirmation of worth. It serves as a seal of approval for a particular method in a sub-discipline or field of study. This praiseworthiness interpretation prompted Claassen (2005) to caution against using the term “gold standard” in absolutist and perfectionistic terms, as if it were an endpoint. On the one hand, Claassen reminded us that “gold standard” originated as a measure of a currency’s worth compared to gold, not as a gold medal for a best or even unsurpassable standard. On the other hand, the gold standard in many fields in English-language use is straightforward exemplary praise.

2. As excellence. Proponents of some quantitative ones over methods commonly subsumed as “other” or of inferior quality have sometimes applied the label gold standard to their social science methodologies. Qualitative methods (e.g., for observational studies) fit this description of “other inferior methods,” although they are more commonly now research approaches in their own right. Critics of heavily quantitative methods cite implementation, statistical, and programmatic failures in experimental research that ignore differences in population, purpose, and cultural context. The phrase gold standard has been used injudiciously to describe excellence in quantitative approaches.

3. As aspiration. This usage is subtler, but some observers have seen it in applications of the PCL-R and the DSM-5, as well as the CRISPR gene-editing technique, criticized for extensive off-target effects (Evers et al., 2014). The conceptual use of gold standard as a target for research and measurement excellence is an infrequent but more appropriate application.

4. As marketing device. The preceding discussion reported using the term gold standard to market the Reiss Motivation Profile of Developmental Disabilities. The descriptor occurs in many other marketing contexts in which the product name includes the term “gold standard.” These applications include Gold Standard Whey, Gold Standard Global Goals, Gold Standard Rewards for bank debit cards, and Gold Standard production of shanks and screws. With such broad usage, the gold standard is in jeopardy of overuse, edging to triteness. The popularized view of gold standard in terms of exceptionalism is contrary to the scientific method.

5. As a simplistic concept, that impedes flexible cognitions. One does not read much specifically about the quality of so-called gold standards in psychology and other social sciences. There are no descriptions comparable to 18 karat (or 75% pure gold) or 24-karat pure gold, the latter of which is too soft for everyday use. Nickel, zinc, silver and various alloys are ways to improve the usability and durability of metals. The same need for multiple ways of considering quality of gold for any practical purpose appears applicable to the social sciences. Claassen’s (2005) perspective seeks a return to a conceptualization of the gold standard as “the best tool available at the present time” instead of “a level of perfection that can never be attained […]” (p. 1121).

This article has addressed why scholars should not continue to use the phrase “the gold standard” to describe best practices or rigorous social science. A fundamental reason for skepticism is that a one-size-fits-all standard of excellence stymies good research. After all, if the field is already using gold, why keep looking? Perhaps a more compelling caution is that the phrase invokes a false sense of validity that is misleading for the researcher, the practitioner, and the layperson alike. Those problems lead our discussion to consideration of the pyrite principle.

## The Pyrite Principle: a Supplemental Conceptualization

We propose the construct of the pyrite principle to supplement the idea of a gold standard in conceptualizing research and practice. This principle would help to address the presumptuousness of the “one way to perfection” interpretation. Pyrite is iron sulfide, the most common of the many sulfides, and is also pejoratively known as *false gold* or *fool’s gold*. The label false gold came about because of a superficial similarity to the appearance of gold. Gold is shiny, relatively soft, and bright yellow. False gold has a history of being mistaken for real gold because of its brassy yellow color and metallic luster. It is false in the sense of having not real value in currency markets. However, pyrite has a long history of being a profoundly useful mineral in human development and contemporary applications. In his definitive volume on pyrite, Rickard (2015) starts with observing its essential role in fire lighting in ancient civilizations and providing material for cave paintings. He goes on to develop in depth the role of pyrite crystals and a core material for many current scientific breakthroughs. He describes how pyrite has played an essential role in the development of crystallography and how the sulfur from pyrite is used in a remarkable range of applications, from the modern arms industry to metal extraction, and from the electronics industry to the study of free radicals.

Why consider a pyrite principle as a supplemental way of thinking about standards, instead of, say, the silver standard? The answer is that pyrite is already linked linguistically and visually to gold; even with the unbecoming label of fool’s good, it is a useful and meaningful mineral in a wide variety of contexts.

We are not proposing that, in research publications, scholars should state that the methods or measures have attained a pyrite standard. That would open up a marked possibility for ridicule. However, there are cogent reasons to include this principle in thinking about excellence in research methods and social science literature. First, the pyrite principle does not use hyperbole as a core concern. It eliminates surplus meanings associated with the word “gold.” The fact that pyrite is referred to as false gold promotes a sense of humility in its use. In a parallel sense, the understanding of social science constructs is imperfect. The worth of these constructs diminishes with methodological overreaches, implicit biases, and loss of social and cultural acuity. Including the pyrite label in social science thinking indicates awareness of such limitations for cautionary purposes. The pyrite principle seeks to get the social sciences unstuck from overuse of gold standard because it is a simplistic, one-dimensional positivistic frame of reference. In other words, the time might be right for experimental use of the humble pyrite construct in scholarly and professional vocabularies.

For these reasons, a pyrite framing is a potentially useful if modest conceptual addition to behavioral sciences. This modestly useful description would apply to adding its flexibility to discussions of the DSM-5, of research methodologies, and of various positivist interventions. Acknowledging the limitations of these concepts in research places them in a more flexible frame of reference.

## Conclusion

It is reasonable to conclude that there is no gold standard in conceptualizing the use of the term “gold standard,” at both a meta and practical level. No amount of historical, linguistic, or research analysis is likely to stop its use. Indeed, if one extrapolates the linguistic devices that identify levels of credit cards and frequent flyer programs—blue or green, silver, gold, sapphire, platinum, titanium, and so on—onto scientific and medical labeling, the various overstatements may well increase. The task for the critical reader and cautious scientist is to view all such exaggerations with skepticism. It is not that useful methodologies and products are unavailable; it is how much one buys into hyperbolic aspects that is the issue.

## Author Contributions

The initial concept of the pyrite principle was developed by SB as was the history of gold and the gold standard. The literature review tasks were equally divided with SB assuming responsibility for psychological literature and BL assuming responsibility of medical and sociological literature. The comparison of pyrite to gold and the development and application of the pyrite principle as an alternative to the gold standard was undertaken jointly.

## Conflict of Interest

The authors declare that the research was conducted in the absence of any commercial or financial relationships that could be construed as a potential conflict of interest.
